# Signature selection analysis reveals candidate genes associated with production traits in Iranian sheep breeds

**DOI:** 10.1186/s12917-021-03077-4

**Published:** 2021-12-03

**Authors:** Leila Mohamadipoor Saadatabadi, Mohammadreza Mohammadabadi, Zeinab Amiri Ghanatsaman, Olena Babenko, Ruslana Stavetska, Oleksandr Kalashnik, Dmytro Kucher, Oleksandr Kochuk-Yashchenko, Hojjat Asadollahpour Nanaei

**Affiliations:** 1 Department of Animal Science, Shahid Bahonar University of Kerman, Kerman, Iran; 2 Department of Animal Science, Fars Agricultural and Natural Resources Research and Education Center, Agricultural Research, Education & Extension Organization (AREEO), Shiraz, Iran; 3 Department of Animal Science, Bila Tserkva National Agrarian University, Soborna, Bila Tserkva, Kyivska Oblast Ukraine; 4 Department of Animal Science, Sumy National Agrarian University, Sumy, Ukraine; 5 Department of Breeding, Animal Genetics and Biotechnology, Polissia National University, Zhytomyr, Ukraine; 6 College of Animal Science and Technology, Northwest A&F University, Yangling, 712100 Shaanxi China

**Keywords:** Candidate genes, Middle east, ovis aries, Positive selection

## Abstract

**Background:**

Sheep were among the first animals to be domesticated. They are raised all over the world and produce a major scale of animal-based protein for human consumption and play an important role in agricultural economy. Iran is one of the important locations for sheep genetic resources in the world. Here, we compared the Illumina Ovine SNP50 BeadChip data of three Iranian local breeds (Moghani, Afshari and Gezel), as a population that does not undergone artificial breeding programs as yet, and five other sheep breeds namely East Friesian white, East Friesian brown, Lacaune, DorsetHorn and Texel to detect genetic mechanisms underlying economical traits and daptation to harsh environments in sheep.

**Results:**

To identify genomic regions that have been targeted by positive selection, we used fixation index (Fst) and nucleotide diversity (Pi) statistics. Further analysis indicated candidate genes involved in different important traits such as; wool production included crimp of wool (*PTPN3, NBEA* and *KRTAP20–2* genes), fiber diameter (*PIK3R4* gene), hair follicle development (*LHX2* gene), the growth and development of fiber (*COL17A1* gene)), adaptation to hot arid environments (*CORIN* gene), adaptive in deficit water status (*CPQ* gene), heat stress (*PLCB4, FAM107B, NBEA, PIK3C2B* and *USP43* genes) in sheep.

**Conclusions:**

We detected several candidate genes related to wool production traits and adaptation to hot arid environments in sheep that can be applicable for inbreeding goals. Our findings not only include the results of previous researches, but also identify a number of novel candidate genes related to studied traits. However, more works will be essential to acknowledge phenotype- genotype relationships of the identified genes in our study.

**Supplementary Information:**

The online version contains supplementary material available at 10.1186/s12917-021-03077-4.

## Background

Small ruminants, especially domestic sheep (*Ovis aries*), have an vital role in the livelihood of a significant portion of human population in the developing and under developing countries [1, 2]. They probably domesticated about 11,000 years before present (BP) from Asian Mouflon (*Ovis orientalis*) in the Fertile cresecent, possibly southeast Anatolia and/or the Zagros [3]. After domestication, geographical isolations and artificial selection have led to substantial variation in the phenotypic traits of different sheep breeds. Today, they are raised all over the world and adaptable to different geographical climates due to their adaptability to low nutrition diets, endurance in intolerable climatic situations and manageable size [4, 5]. Around 44.9% of the world’s sheep populations are live in Asian continent [6], and Iran has the largest sheep population of 52 million in the Middle East. More than 27 sheep ecotypes have been recognized in Iran which are vary in different factors, including their genetic potential for milk, meat and wool production traits [7, 8]. These breeds are conventionally named in accordance with their geographical origin, also thay have categorized according to productive performance and morphological features [9–12]. Despite lower production efficiency of Iranian local sheep population than commercial dairy and meat breeds, they have adapted to various evolutionary trajectory based on genetic drift and regional adaptation, and have not been undergone artificial breeding programs as yet [13]. For example, Moghani, Afshari and Gezel are three well-known dual-purpose sheep breeds in Iran. The Ghezel is one of the native fat-tailed breeds in the north-western part of Iran, dual purpose (meal and milk). The Afshari is one the fat-tailed carpet wool sheep breeds, known for its large size, that has been originated from north-west of Iran (Zanjan province). The Moghani sheep is one of the medium-size Iranian fat-tailed sheep breeds, which can be found in the West Azerbaijan province. This breed is good for both the wool and meat production traits.

A considerable diversity in production [14] and daptation [15, 16] traits were detected across and within different sheep breeds [14]. Iran is placed in the Africa-Asia girdle with 90% of arid regions, which is considered as a hot and arid country in terms of climate in the world [17, 18]. Conserving these diversity is important for raising the efficiency of production and imporoving adaptation to environments. Until now, different studies have been done to identify genes related to production and daptation traits in sheep using methods such as methylated DNA immunoprecipitation sequencing [19–21], RNA-Seq analyses [22–27], whole genome sequencing nalyses [28, 29] and genome-wide association studies [30–33]. Although Iranian indigenous sheep breeds display high genetic potential for production, researches to increase their productivity is less carried out compared to other sheep breeds. There are only a few genome-wide association [34–37] and signatures selection mapping [38] studies related to some Iranian sheep breeds.

In this research, we used the fixation index (FST), as a measure of population differentiation due to genetic structur and nucleotide diversity (Pi), to compare the Illumina Ovine SNP50 BeadChip data of three Iranian local breeds (Moghani, Afshari and Gezel) with those of the dairy (East Friesian white, East Friesian brown and Lacaune) and meat (DorsetHorn and Texel) sheep breeds to explore novel candidate genes related to economic and adaptation traits in Iranian local sheep breeds.

## Results

### Population genetic structure

**Fig. 1:**
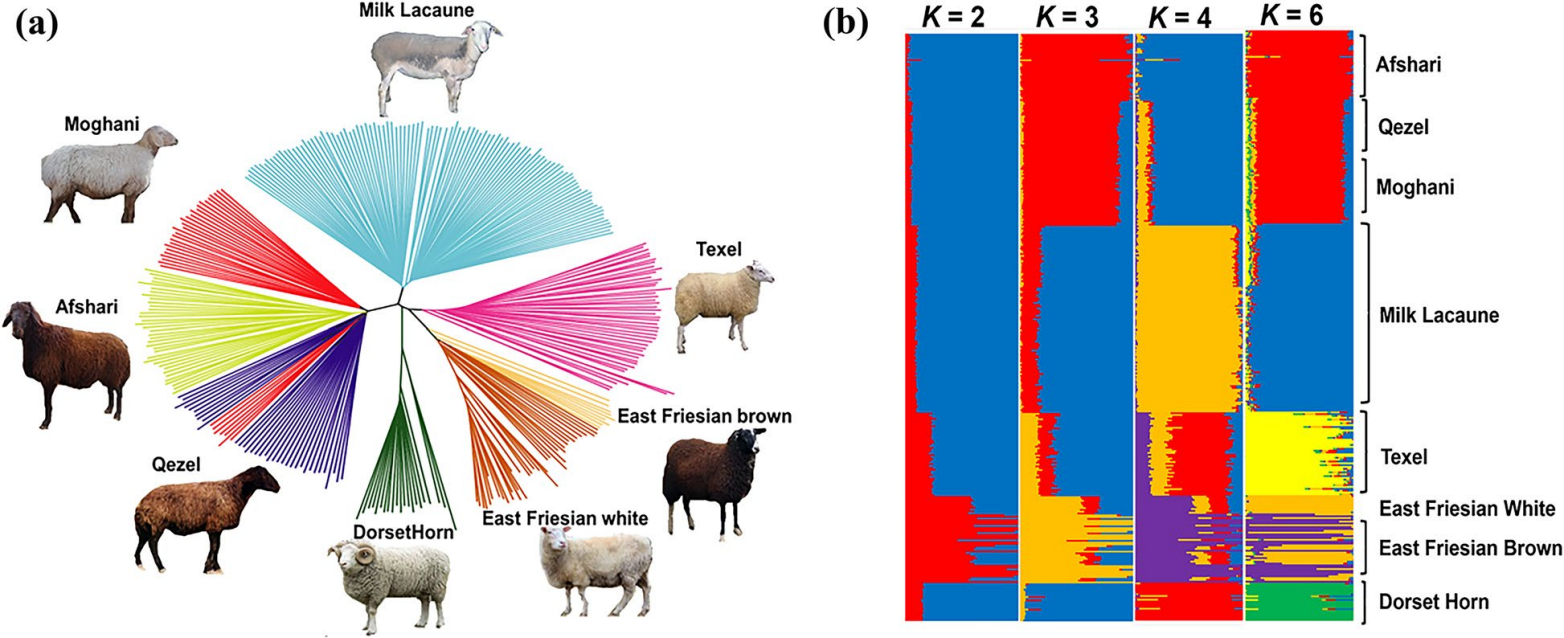
Neighbour-joining (NJ) tree (**a**) and Output of Admixture (**b**) for k values of 1–6, the best k value is 2

A total of 324 individuals and 43,586 SNPs were selected for downstream analysis. After applying LD pruning, SNPs were used for ADMIXTURE analysis. The genetic distances tree showed that all three Iranian local sheep breeds shared a common clade and all other commercial breeds were placed into separated clades (Fig. 1a). To more study the relationships gained from phylogenetic analysis, we carry outed Admixture analysis based on a Bayesian model for different values of K (*k* = 2–6). We further found a clear differentiation between Iranian local sheep samples and other groups from *K* values in 3–6 (Fig. 1b).

**Fig. 2:**
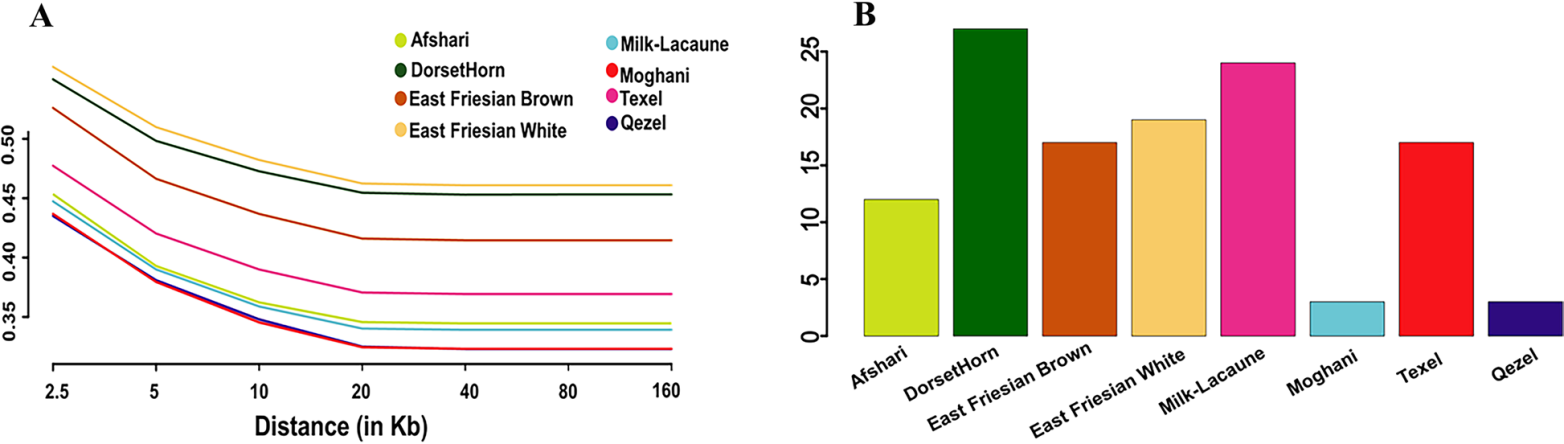
LD decay (**A**). Average number of ROHs longer than 1000 Kb for each population (**B**)

The results collectively showed that all three Iranian local sheep populations may have endured different evolutionary **processes** in accordance with their genetic breeding history and regional adaptation/selection following domestication. We then estimated the patterns of LD, which can be informative for different biological and historical factors including, recombination, selection, inbreeding, and population bottlenecks. The LD pattern showed that the mean of r^2^ in Iranian breeds was less than 0.5 in window different sizes (2.5, 5, 10, 20, 40, 80 and 160 Kb). The amount of LD was the highest in the East Friesian and DorsetHorn and the lowest in Moghani and Qezel respectively (Fig. 2A). In addition, we calculated runs of homozygosity (ROH) to assess recent inbreeding. The distribution of ROHs for all studied sheep breeds are shown in Fig. 2B. Here we found a markedly higher number of ROHs in DorsetHorn, Milk Lacaune and East Friesian whitegenomes than other sheep breeds, that could be a consequence of artificial selection for traits of interest, e.g. milk and meat production traits, in thire breeding programs. Also, whithin Iranian sheep breedsboth Moghani and Qezel breeds had the lowest number of ROHs (Fig. 2b). The results showing the less recent inbreeding in moghani and Qezel compared with that in the other breeds.

### Genome-wide selective sweep analysis

**Fig. 3:**
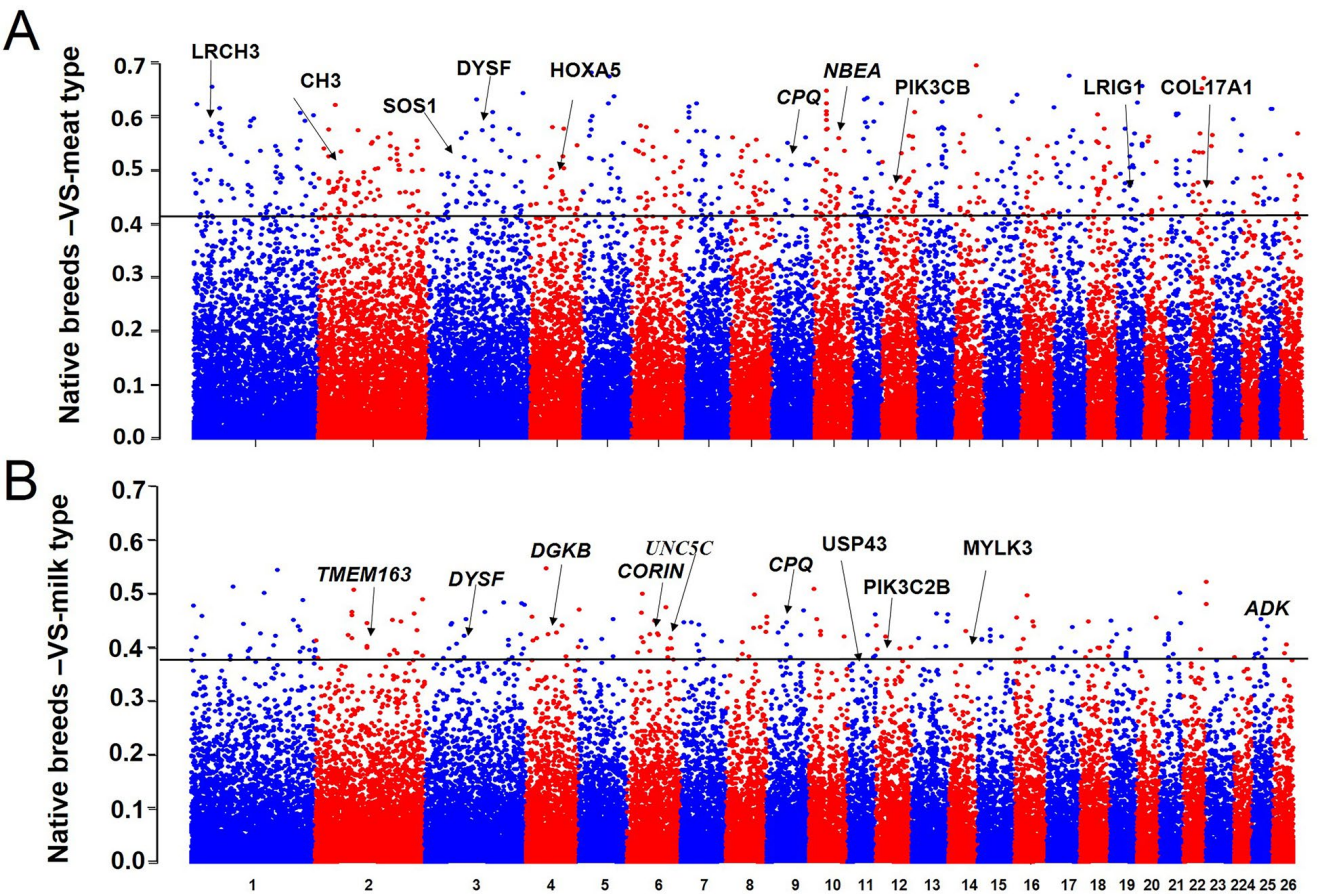
Manhattan plot of genome-wide Fst values. **A** Indigenous group versus meat group. **B** Indigenous group versus milk group

In this study we used Ovine SNP50 BeadChip data for comparative genome analysis between Iranian sheep breeds (Moghani, Afshari and Gezel) and different commercial populations (dairy and meat types) to identify regions in the genome that are associated with different phenotypes. We applied FST nucleotide diversity (Pi) statistics to detect genomic footprints left by natural selection in local sheep breeds. The regions having lower levels of nucleotide diversity and extremely high FST values (top 1 and 5%) were studied to be regions potentially under selection. A number of genes that covers significant nucleotide diversity and FST values (Fig. 3 and supplementary file 1: Table S1) were identified in different comparations. To examine the functional complexity of each gene set, gene enrichment was performed on the gene lists produced by different comparations. Gene set enrichment analysis (GSEA) identified enriched categories related with “biological process”, “human phenotype” and “molecular function” (supplementary file 2: Table S2, supplementary file 3: Table S3, supplementary file 4: Table 4 and supplementary file 5: Table S5).

## Discussion

We firstly applied different population genomic analyses to reveal genetic relationships among different sheep populations (Figs. 1 and 2). The phylogenetic distance based on the whole-genome information showed all Iranian native breeds have a obviously distinict genetic distance from the other commercial groups. Also Iranian sheep breeds were seen as a dependent group in admixture output (k values of 3–6). The ROHs and LD values represent the decrising of recent inbreeding in Iranian local sheep breeds due to no artificial selection compared with other commercial sheep breeds.

Our results collectively showed that all three Iranian local sheep breeds may have experience different evolutionary path based on genetic drift and various regional adaptation after domestication, which is in agreement with previous studies [8, 39, 40]. We then detected a number of candidate genes that previously have reported associated with production traits in sheep and other livestock species. The significant candidate genes related to wool trait and daptation to harsh and hot arid environments are summerized in supplementary file 1: Table S1.

In the following, only main candidate genes are mentioned below to discuss their potential involvements in controlling wool production traits and adaptation to hot arid environments.

### Adaptation and genes associated with heat stress, hot arid and harsh environments

Heat stress has a negative effect on the biological functions of sheep and disrupts their production, fertility, and health characteristics [41]. Maintaining animal performance in hot climates can be achieved by identifying genetic exclusivity associated with heat tolerance [42]. Recent studies showed that functional genes are present in epigenetic thermoregulatory mechanisms that lead to adaptive behavioral and protective responses [43].

Phosphatidylinositol-4-phosphate 3-kinase catalytic subunit type 2 beta (*PIK3C2B*) gene encodes phosphatidylinositol-4-phosphate 3-kinase catalytic subunit type 2 beta and is located on sheep chromosome 12. In this research, *PIK3C2B* gene is considered as a region under selection through comparing Iranian native populations breeds with both commercial dairy and meat sheep breeds (top 5% for FST). A study has detected that genes enriched in the type II diabetes mellitus pathway including phosphoinositide 3-kinase genes are responsible for responding to adaptation mechanisms. These genes are responsible for many biological functions in the intracellular signal transduction pathways, such as immunity, metabolic control, and cardiovascular homeostasis. The *PIK3C2B* gene is grouped in a cluster of genes compatible with PI3K isoforms that cause energy homeostasis in response to relatively high energy demand under heat stress conditions and has an important relationship with adaptation mechanisms to heat stress in ducks [44].

Phospholipase C beta 4 (*PLCB4*) gene encodes phospholipase C beta 4, was found in one of the selected regions on Chr. 13, between Iranian local sheep breeds with commercial dairy sheep breeds (top 5% for pi). Li et al. [45] have reported that *PLCB4* gene is correlated with heat tolerance (oxidative stress response) in Dehong humped cattle. Also, Jin et al. [46], by studying of the catfish genome, showed that this gene is related to heat stress (energy metabolism) in aquatic species [46].

Another gene that responds to changes in heat stress levels is *FAM107B* (family with sequence similarity 107 member B) gene that was situated in one of the selected regions on Chr. 13, between Iranian native sheep breeds with commercial dairy sheep breeds (top 5% for pi). This gene plays a role in heat-shock induction [47]. A recent genome-wide association study in Holstein cattle has shown that the *FAM107B* gene is strongly associated with heat stress response in dairy cattle [48]. Also, *USP43* (ubiquitin specific peptidase 43, Chr. 11 sheep, comparing between Iranian local sheep breeds and commercial dairy sheep breeds (top 5% for FST)) gene is involved in heat stress through the ubiquitination process, and its expression was increased in skeletal muscle lambs exposed to heat stress [49].

*CORIN* (corin, serine peptidase, Chr. 6 sheep, comparing between Iranian local sheep breeds commercial dairy sheep breeds (top 5% for FST)) gene encodes a serine proteinase that is involved in activating proteins related to volume and blood pressure [50, 51]. This gene is involved in the circulatory system and maintaining the proper volume of blood, so can play an important role in adapting to temperature regulation in hot and dry environments [52].

Carboxypeptidase Q (*CPQ*) gene encodes carboxypeptidase Q is located on the sheep chromosome 9. This gene is regarded as a region under selection through comparing Iranian local sheep breeds with both commercial dairy and meat sheep breeds (top 5% for FST) and Iranian native populations breeds with commercial dairy sheep breeds (top 1% for Pi). *CPQ* Gene is involved in proteolytic functions. Degradation of protein in plants, which depends on the levels of proteolytic enzymes, is an important part of the plant’s response to environmental stress. Drought affects on the function of these enzymes so that it leads to significant changes in proteolytic activity levels in plant leaves, which can be part of the drought-resistance mechanism [53]. accordingly, Almas et al., in a study of African indigenous chickens, introduced this gene as an important adaptive gene in deficit water status [54].

*Neurobeachin* (NBEA) gene encodes neurobeachin, was found in one of the selected regions on Chr. 10, between Iranian local sheep breeds with commercial meat sheep breeds (top 5% for FST and top 1% for Pi). *NBEA gene* has a relationship with regulating body temperature in cattle within heat stress [55] and also to contribute feed intake and body weight [56].

### Genes associated with the wool trait

Wool is a valuable natural fiber that varies in crimp, elasticity, and diameter, and its quality affects the economic performance of wool sheep [57]. Carpet wool is developed by the thickening and elongation of the wool module in the primary wool follicles [57].

*COL17A1* (collagen type XVII alpha 1 chain, Chr. 22) candidate gene related to coarse wool, was found between Iranian local sheep breeds with commercial meat sheep breeds (top 5% for FST). Nie et al. [58] Using transcriptome long non-coding RNAs (LncRNAs), and mRNAs involved in the excitation of the primary wool follicle in the skin of carpet embryo sheep, have reported that there is an intricate regulatory relation between LncRNAs and mRNAs in the formation of primary follicles. *COL17A1* was one of the genes identified in their research in which mRNA of *COL17A1* gene regulates the dermal-epidermal junction and basement membrane, and lncRNAs of *COL17A1* gene are relevant to collagens [58].

Neurobeachin (*NBEA*) gene encodes neurobeachin, was found in one of the selected regions on Chr. 10, between Iranian local sheep breeds with commercial meat sheep breeds (top 5% for FST and top 1% for Pi). Studies show that the function of this gene is related to epithelial cells or skin development. Wang et al. [59] have reported that the *NBEA* gene is related to the crimp trait in the Chinese Merino sheep. In the current study, *PIK3R4* (phosphoinositide-3-kinase, regulatory subunit 4) gene s located on Chr. 1 sheep, between Iranian local sheep breeds with commercial milk sheep breeds (top 5% for pi) and was identified as a gene related to wool diameter [59].

Another gene related to wool, *KRTAP20–2* (keratin-associated protein 20–2) gene is located on Chr. 1 sheep, between Iranian local sheep breeds with commercial milk sheep breeds (top 5% for pi). Studies have shown that SNPs in *the KRTAP20–2* gene are associated with cashmere weight and length in goats [60] and wool fiber curvature in sheep [61].

Another wool-related gene, protein tyrosine phosphatase non-receptor type 3 (*PTPN3*), was found in one of the selected regions on Chr. 2, between Iranian local sheep breeds with both commercial dairy and meat sheep breeds (top 1% for Pi). *PTPN3* gene is a member of the FERM proteins family (4.1/ezrin/radixin/moesin). The *PTPN3* gene product is a protein phosphatase and a structural component of the cytoskeleton that plays an important role in the maintenance of tight junction integrity between the cell membrane and the cytoskeleton. The *PTPN3* gene product is associated with focal adhesions [62–64]. One previous study has reported that *PTPN3* gene is associated with crimp traits in the wool of Chinese Merino sheep [59].

LIM homeobox 2 (*LHX2*) an encoding gene is located on Chr. 3, between Iranian local sheep breeds and commercial meat sheep breeds (top 1% for Pi). It is a flexible and hydrophobic protein and is involved in secondary hair follicle development [65] and studies have shown that *LHX2* gene is not expressed in the resting phase of hair follicles but is active in their growth phase [66], this gene helps to maintain stem cell-related properties in hair follicles [67].

## Conclusions

We detected several novel candidate genes related with wool production traits and adaptation to hot arid and harsh environments in sheep that can be applicable for inbreeding goals. However, more works will be essential to acknowledge phenotype- genotype relationships of the identified genes in our study.

## Materials and methods

### Population data and data preprocessing

The Illumina Ovine SNP50 BeadChip data of 324 sheep individuals, including 106 indigenous Iranian breeds (Moghani *n* = 34, Afshari *n* = 37 and Gezel *n* = 35) and four commercial breeds (DorsetHorn *n* = 21, Texel *n* = 46, Milk Lacaune *n* = 103, East Friesian white *n* = 9 and East Friesian brown *n* = 39) were obtained from the Sheep HapMap project database (Data Collection. 10.4225/08/51870B1E8EE56) [68]. The SNP quality control (QC) was performed by plink 1.07 program [69]. All SNPs were filtered based on minor allele frequency (maf > 0.01), call rate less than 90% and Hardy–Weinberg equilibrium (hwe > 10^− 5^).

### Population structure, ROH and LD decay

To reduce the powerful influence of SNPs clusters in relatedness analysis, PLINK was used to prune the filtered SNPs (indep-pairwise 100 50 0.1). Admixture tool [70] was applied for visualize population structure in our sheep samples, with an ancestor population size ranging from 2 to 6 and 10,000 iterations for each run, based on the pruned data. Amounts ROHs (−-homozyg-kb and --homozyg-snp) lengths > 100 Kb and LD (with –r2 flag) for seven distance classes (2.5, 5, 10, 20, 40, 80 and 160 kb) were also computed using PLINK software. Genetic relationships among the individuals was investigated based on neighbor joining approach with pair-wide distances. Genetic distances tree was drawn using bootstrap method (No. of Bootstrap Replications, 1000) in MEGA7.

### Signatures of positive selection and annotation

Recently different techniques have been used to reveal signatures of positive selection in genomes [71]. F_ST_ statistics is the most extensively applied and the strongest method that benefit from the variation across the genome [72, 73]. In this study in order to detect novel candidate genes included in genetic traits under selection, F_ST_ and nucleotide diversity (Pi) statistics were used to compare the SNP data of the Iranian breeds with those of dairy and meat breeds. Here, to identify the genomic regions under positive selection in eight sheep breeds, we detected the “outlier” loci with locus-specific FST estimates, as presented by Akey et al., (2002) as follows:

$${F}_{ST}=\frac{MSP- MSG}{MSP+\left({n}_c-1\right)\ MSG}$$

Here, MSP shows the observed mean square errors for Iranian sheep population and other sheep populations,

$$MSP=\frac1{S-1}\;{\textstyle\sum_i^s}n_i\left(p_{Ai}-{\overline P}_A\right)^2$$

Where, MSG shows the observed mean square errors for loci within sheep populations.

$$\mathrm{MSG}=\frac{1}{\sum_{i=1}^S{n}_i-1}\ \sum_i^s{n}_i{p}_{Ai}\left(1-{p}_{Ai}\right)$$

In the above phrase, *i* shows the subpopulations (where *i* = 1, …, s), *ni* the sample size in subpopulation *i*, *P*_*Ai*_ the frequency of the SNP allele *A* in the *i*th subpopulation, *n*_*c*_ is the mean sample size across samples [74], and

$$\overline{P}={n}_i{p}_{Ai}/{\sum}_i{n}_i.$$

A sliding window method (100 kb with a step size of 50 kb) was applied to compute pairwise Fst using VCFtools 0.1.15 (http://vcftools.sourceforge.net/index.html) [75] and level of significance at cut off *P* ≤ 0.05 was used for FST values.

The nucleotide diversity was calculated as the proportion of pairwise differences between two populations, (π(other sheep populations)- π(Iranian sheep population)), finally the top 1% was separated as positively selected regions. The nucleotide diversity (π) was calculated using a step size of 25 kb and window size of 50 kb. To assist following gene annotation, the UCSC liftOver tool [76] (https://genome.ucsc.edu/cgi-bin/hgLiftOver) was applied to upgrade the all genomic positions to accordance the sheep genome Oar_v4.0. Sheep gene IDs that involved selected genomic regions were obtained from Ensemble annotation. Gene ontology (GO) analysis was carried out in g:Profiler toolset [77] using Benjamini–Hochberg false discovery rate procedure and we only reported the significant terms (*P*-value < 0.05).

## Supplementary Information

**Additional file 1: Table S1.** Candidate genes related to production traits identified through signature selection analysis in sheep.

**Additional file 2: Table S2.** Gene enrichment among regions under selection through compare the SNP data of the Iranian breeds with those of meat breeds using Fst method.

**Additional file 3: Table S3.** Gene enrichment among regions under selection through compare the SNP data of the Iranian breeds with those of dairy breeds using Fst method.

**Additional file 4: Table S4.** Gene enrichment among regions under selection through compare the SNP data of the Iranian breeds with those of meat breeds using nucleotide diversity (Pi).

**Additional file 5: Table S5.** Gene enrichment among regions under selection through compare the SNP data of the Iranian breeds with those of dairy breeds using nucleotide diversity (Pi).

## Data Availability

The data that support the findings of this study are available from the corresponding author upon reasonable request.

## Abbreviations

COL17A1: Collagen type XVII alpha 1
CORIN: Corin, serine peptidase
CPQ: Carboxypeptidase Q
FAM107B: Family with sequence similarity 107 member B
FST: Fixation index
GO: Gene ontology
KRTAP20–2: Keratin-associated protein 20–2
LD: Linkage disequilibrium
LHX2: LIM homeobox 2
LncRNAs: Long non-coding RNAs
MAF: Minor allele frequency
NBEA: Neurobeachin
PIK3C2B: Phosphatidylinositol-4-phosphate 3-kinase catalytic subunit type 2 beta
PIK3R4: Phosphoinositide-3-kinase, regulatory subunit 4
PLCB4: Phospholipase C beta 4
PTPN3: Protein tyrosine phosphatase non-receptor type 3
ROHs: Runs of homozygosity.
USP43: Ubiquitin specific peptidase 43
