# Supplementary material for: Signature selection analysis reveals candidate genes associated with production traits in Iranian sheep breeds
Source: BMC Vet Res. 2021 Dec 3;17:369. doi: 10.1186/s12917-021-03077-4 (PMC8641187; doi:10.1186/s12917-021-03077-4)
Supplement: Supplementary file 1 — Additional file 1: Table S1. Candidate genes related to production traits identified through signature selection analysis in sheep. [file 12917_2021_3077_MOESM1_ESM.docx]

Table S 1 Candidate genes related to production traits identified through signature selection analysis in sheep

| Method | Trait | Gene | Chr. | Summary of gene function |
| --- | --- | --- | --- | --- |
| FST (between Iranian  native sheep breeds and commercial dairy sheep breeds) (top 5%) | Immunity | ADK | 25 | Immunogenicity and protective response against a virulent strain B. abortus 544 infection (Huy et al. 2020) |
|  | adaptation of hot arid and harsh environment | CORIN | 6 | Through circulatory system and blood vessel development (Gu et al. 2020) |
|  | Adaptive mechanism to the water deficit condition | CPQ | 9 | Adaptive mechanism to the water deficit condition (Gheyas et al. 2020) |
|  | immunity in mammals. | DGKB | chr4 | resistance to gastrointestinal parasites (Zvinorova et al. 2017) |
|  | adaptation | DYSF | [assembly 3](https://asia.ensembl.org/Ovis_aries_rambouillet/Location/View?db=core;g=ENSOARG00020005927;r=3:99648330-99873763) | high-altitude adaptation (Wei et al.2016) |
|  | adaptation | FZD6 | 9 | associated with high altitude hypoxia adaptation (Liu et al. 2020). |
|  | disease resistance | MYLK3 | 14 | disease resistance traits,  this gene plays key roles in resistance to diarrhea  (Xu et al. 2019) |
|  | adaptation | GRIN2B | 3 | high altitude adaptation  oxygen intake (Ai et al. 2014) |
|  | adaptation to heat stress | PIK3C2B | 12 | adaptation to heat stress (Kim et al. 2017) |
|  | Wool growth | TMEM163 | 2 | zinc transporter (Styrpejko et al. 2020; Cantrell 2016) |
|  | hair follicle fate | UNC5C | 6 | Involved in β-catenin signaling program that is required for hair follicle development (as one of potential mediators of the effects of β-catenin signaling on pigmentation and innervation) (Zhang et al. 2008). |
|  | acclimation to the environment | USP43 | 11 | acclimation to the environment  (Heat stress) (Kubik et al., 2018) |
|  | susceptible to infections with Escherichia col | LRCH3 | 1 | susceptible to infections with Escherichia coli (E. coli) or infection of the small intestine by enterotoxigenic Escherichia coli  (Jacobsen et al., 2010) and  higher susceptibility to Mycobacterium avium ssp. paratuberculosis infection (McGovern et al. 2019, Rivière et al. 2020) |
| FST (between Iranian native sheep breeds and commercial meat sheep breeds (top 5%) | adaptation | EDNRB | 10 | High-altitude adaptation  Through cardiac tolerance  (Azad et al. 2017) |
|  | adaptation | DYSF | [assembly 3](https://asia.ensembl.org/Ovis_aries_rambouillet/Location/View?db=core;g=ENSOARG00020005927;r=3:99648330-99873763) | high-altitude adaptation (Wei et al.2016) |
|  | wool | COL17A1 | 22 | dermal-epidermal junction and basement membrane (Nie et al. 2018), collagens  (Gong et al., 2018; McGrath et al. 1996) and the growth and development of fiber (Barazandeh et al. 2020) |
|  | wool growth | HOXA5 | 4 | associated with anagen wool growth (Zhao et al.2017) |
|  | Wool | LRIG1 | 19 | Hair Follicle (et al. 2017; Gong et al; 2020) |
|  | Wool | NBEA | 10 | crimp trait (Wang et al. 2014)  also  associated with support of body temperature in cattle during heat stress (Howard et al., 2014;) and  also known to contribute to body weight and feed intake (Olszewski et al. 2012) |
|  | heat tolerance | SHC3 | 2 | linked to the residual feed intak (Li et al. 2020) |
|  | hair follicle | SOS1 | 3 | hair follicle (Liceras-Boillos et al. 2018) |
|  | high-altitude adaptation | TACR1 | 3 | high-altitude adaptation (Edea et al.2019 ) |
|  | adaptation | DYSF | [assembly 3](https://asia.ensembl.org/Ovis_aries_rambouillet/Location/View?db=core;g=ENSOARG00020005927;r=3:99648330-99873763) | high-altitude adaptation (Wei et al.2016) |
|  | susceptibility to disease | LRCH3 | 1 | susceptible to infections with Escherichia coli (E. coli) or nfection of the small intestine by enterotoxigenic Escherichia coli  (Jacobsen et al., 2010) and  higher susceptibility to Mycobacterium avium ssp. paratuberculosis infection (McGovern et al. 2019, Rivière et al. 2020) |
|  | Adaptive mechanism to the water deficit condition | CPQ | 9 | Adaptive mechanism to the water deficit condition (Gheyas et al. 2020) |
|  | adaptation to heat stress | PIK3C2B | 12 | adaptation to heat stress (Kim et al. 2017) |
| Pi (between Iranian native sheep breeds and commercial milk sheep breeds (top 1%) | Adaptive mechanism to the water deficit condition | CPQ | 9 | Adaptive mechanism to the water deficit condition (Gheyas et al. 2020) |
|  | Hair Follicle | COL6A6 | 1 | Hair Follicle (Yue et al., 2016) |
|  | Coat color | DOCK8 | 2 | Coat color (Saif et al. 2021) |
|  | coat coloring pattern | ERBB4 | 2 | coat coloring pattern (Qanbari et al. 2014) |
|  | adaptation | FAM107B | 13 | heat stress (Luo et al. 2021) |
|  | Adaptation | GALNT16 | 7 | Adaptation to Tropical Forests  (Amorim et al. 2015) |
|  | response to stress and in immune system | GPS2 | 11 | involved in response to stress and in immune system processes. and  involved in the development of leukemia in humans(Casas et al. 2020) |
|  | wool | KRT5 | 3 | hair/fleece development and functionand keratin associated protein  (Kang et al. 2013) |
|  | wool | KRTAP20-2 | 1 | wool fiber curvature  (Gong et al. 2019) |
|  | hair follicles | LRRC15 | 1 | hair follicles keratinisation and hair shaft differentiation (Gao et al. 2016) |
|  | immune response | LY96 | 9 | immune response (Mohammadi et al., 2018) |
|  | brucellosis resistance | NLGN1 | 1 | brucellosis resistance  (Li et al. 2021) |
|  | wool | PIK3R4 | 1 | Fiber diameter  (Wang et al.2014) |
|  | heat stress | PLCB4 | 13 | heat stress, genes directly involved in thermal tolerance (Li et al.2020), heat stress-associated through  energy metabolism (Jin et al. 2017) |
|  | wool | PTPN3 | 2 | Crimp of wool  (Wang et al. 2014) |
| Pi (between Iranian native sheep breeds and commercial meat sheep breeds (top 1%) | Heat stress | CEP170 | 12 | Rectal Temperature during Heat Stress  (Dikmen, et al.2013) |
|  | wool | COL21A1 | 20 | Greasy fleece weight  (Ebrahimi, et al. 2017) |
|  | Adaptation | DOK1 | 3 | Adaptation to cool environment  (Uchida et al. 2010) |
|  | adaptation | DYSF | [assembly 3](https://asia.ensembl.org/Ovis_aries_rambouillet/Location/View?db=core;g=ENSOARG00020005927;r=3:99648330-99873763) |  |
|  |  | EDNRB | 10 | High-altitude adaptation  Through cardiac tolerance  (Azad et al. 2017) |
|  | wool | LHX2 | 3 | involved in secondary hair follicle development (Geng et al. 2014, Wang et al. 2015) Lhx2 Maintains Stem Cell Character in Hair Follicles (Rhee et al. 2006) |
|  | disease resistance | MYLK | 1 | disease resistance traits  this gene playe key roles in resistance to diarrhea  (Xu et al. 2019) |
|  | wool | NBEA | 10 | Crimp of wool  (Wang et al. 2014)  associated with support of body temperature in cattle during heat stress (Howard et al., 2014; ) and  also known to contribute to body weight and feed intake (Olszewski et al. 2012) |
|  |  |  | 1 | brucellosis resistance  (Li et al. 2021) |
|  | wool | PTPN3 | 2 | Crimp of wool  (Wang et al. 2014) |
|  | coat coloring pattern | ERBB4 | 2 | coat coloring pattern (Qanbari et al. 2014) |
